# Flying Blind: How Thorough are IRBs when Assessing Scientific Value?

**DOI:** 10.1007/s11606-024-09286-5

**Published:** 2024-12-20

**Authors:** Carol Shum, Spencer Phillips Hey, Michael S. Wilkes, John A. Powers, Melissa Ann Pighin, Mark Yarborough

**Affiliations:** 1 Department of Public Health Sciences, University of California Davis, Davis, USA; 2 Prism Analytic Technologies, Boston, MA USA; 3 Department of Internal Medicine, General Internal Medicine, University of California Davis, Davis, USA; 4 George Washington University, Washington, USA

**Keywords:** IRB, clinical trials, scientific rigor, ethics

## Abstract

**Background:**

Institutional Review Boards (IRBs) in the United States play a crucial role in ensuring the ethical conduct of clinical trials, including assessing the scientific merit of studies to justify the risks to participants. However, prior research suggests that many IRBs do not systematically evaluate scientific merit, raising concerns about the approval of low-quality trials.

**Objective:**

To investigate whether IRBs provide adequate guidance on assessing scientific merit in their Standard Operating Procedures (SOPs) and other relevant materials.

**Design:**

A systematic pilot investigation of IRB SOPs and related guidance documents from a sample of U.S.-based non-profit institutions.

**Participants:**

IRB materials from 35 U.S.-based non-profit institutions selected from the FDA’s Bioresearch Monitoring Information System database, representing 39.9% of submissions between 2018 and 2021. Additionally, materials from one U.S.-based for-profit IRB were included.

**Interventions:**

Not applicable.

**Main Measures:**

The presence of guidance on 15 dimensions of scientific merit, clustered into four PICO (Population, Intervention, Comparator, Outcome) categories, was assessed by reviewing IRB SOPs and related documents. Evidence of guidance was determined by mention of keywords related to each dimension.

**Key Results:**

Most IRB materials mentioned basic study elements such as study design (99%), subject recruitment (90%), and intervention justification (97%). However, critical aspects related to study quality, such as bias reduction (53%) and outcome measurement tools (57%), were less frequently mentioned. The least represented dimension was confounder control (10%).

**Conclusions:**

IRB guidance materials vary in their coverage of scientific merit dimensions, with significant gaps in areas critical for assessing study quality. Strengthening guidance materials by including comprehensive instructions for all 15 dimensions could improve IRB assessments of scientific merit, thereby enhancing the ethical oversight of clinical trials.

## INTRODUCTION

Rigorous regulatory oversight of human clinical research trials is a necessity. In the United States, Institutional Review Boards (IRB) are a key component of this ethical oversight. The IRB system exists to help ensure the ethical treatment of the volunteers—usually patients—who make clinical trials possible through their volunteerism and altruism. According to the U.S. federal regulations, a cornerstone of the IRB's ethical duty is to ensure that all trials have sufficient scientific merit to offset the risks and burdens of the volunteers.^1^

Unfortunately, studies examining the scientific quality of clinical trials have consistently found that a significant portion of trials are biased^2^ and lacking scientific quality due to, for example, lack of masking or randomization^3^, inappropriate choice of comparators^4^, or poor primary outcome selection.^5^ All these deficiencies in scientific merit can—and should—be detected and prevented by IRBs.^6^

However, direct interviews of U.S. IRB members have also consistently found that they do not systematically evaluate scientific merit. Some IRB members simply admit that they ignore this duty outright.^7^ Others state that they do think scientific merit is important, but that they do not feel qualified to make the assessment.^8^ Further, a 2023 investigation by the U.S. Government Accountability Office (GAO) into IRB oversight found that neither the government regulators in charge of overseeing IRBs nor the IRBs’ home institutions have been diligent enough in checking to see if trial volunteers are having their health and interests protected.^9^

Despite these discouraging signs for the quality (or existence) of scientific merit assessments within IRBs, it seemed possible that some—perhaps many—IRBs do take the duty to ensure scientific merit seriously. But the apparent failure to prevent low-value trials stemmed from an inadequacy in the guidance or training that IRBs receive in how to go about fulfilling this duty. We therefore undertook a systematic, pilot investigation of the IRBs’ Standard Operating Procedures (SOP)/Investigator Manuals to examine what guidance, if any, these documents provided vis-a-vis scientific merit. Our hypothesis was that if IRBs are indeed conducting scientific merit assessments in accord with their ethical duty, there should be evidence of this in their materials provided to their applicants and members.

## METHODS

### Sample of Materials

Using the Bioresearch Monitoring Information System (BMIS) database within the U.S. Food and Drug Administration (FDA) website, we identified the names of 34 non-profit U.S.-based institutions with public IRB materials that had the most submissions between 2018 and 2021. Additionally, we included the University of California—Davis because this was the home institution of the principal investigators. These 35 non-profit US-based institutions represented 39.9% of all submissions to BMIS during this period from verified U.S.-based IRB institutions.

With our list of 35 non-profit institutions, we searched online for each IRB’s sample protocol template, checklists, and other materials including their Standard Operating Procedures (SOP)/Investigator Manual. This goal was to build a comprehensive dossier representing each IRBs review process. For instances where institutions did not have an IRB SOP/Investigator Manual on their IRB website, we reached out for information via email. Additionally, to get a better idea of the IRB review process within for-profit IRBs, we reached out to several prominent U.S.-based for-profit IRBs for their review materials. Only one of these IRBs responded to our request. It provided us with review materials, which included their IRB member handbook along with some marketing material.

### Extraction and Analysis

For each institution, we searched through all of the IRB supporting materials to look for terms that would indicate awareness and potential guidance for 15 different dimensions of scientific merit (see Table 1). We selected 15 dimensions around which there is consensus in the ethics and scientific literature that the dimension is important for the scientific merit of a trial. We also attempted to select a set of dimensions relevant to different aspects of a study protocol, using the gold-standard systematic review “PICO” categories of Population, Intervention, Comparator, and Outcome to cluster the dimensions.

**Table 1:** Results of PICO-element Keyword Matching in IRB Guidance Materials

Dimension of Scientific Merit	Keywords searched	Any keyword found (N = 35)	%
**Population Elements**			
Study design	design, procedure	33.5	99
Study location	location, site, region	29	84
Study duration	duration, length, visit	29	86
Subject recruitment	recruitment, inclusion, exclusion	30.5	90
Diversity inclusion	race, ethnicity, diversity, minority	26.5	79
Confounder control	confound, subgroup, cohort, stratif*	3.5	10
**Intervention Elements**			
Intervention justification	novel, biological effect, benef*	33	97
Intervention administration	dosage, dose, administer, regimen	21.5	61
Intervention risk assessment	risk, adverse, complication	33	97
**Comparator Elements**			
Comparative treatment	placebo, treatment, arm, control, intervention	23.5	70
Randomization	arm, randomize, ratio	22	63
Bias reduction	blind, open-label	18.5	53
**Outcome Elements**			
Primary/secondary measures or endpoints	primary/secondary endpoint, primary/secondary objective, primary/secondary outcome	24	71
Outcome measurement tools	efficacy, measur*	19	57
Follow-up periods	follow-up	23	66
Subject withdrawal	withdrawal, termination, drop out	27	80

IRB materials from 35 different institutions were systematically searched for keywords relevant to these 15 dimensions of scientific merit. The raw counts for each element are shown in the “Any keyword found” column. Keywords with a “*” were wildcard searches that would match against any word with the corresponding stem (e.g., “stratif*” would match against “stratified” or “stratification”)

Two reviewers reviewed all guidance materials. If an IRB document merely mentioned words or phrases relevant to a dimension of scientific merit, this was considered to be positive evidence (and scored 1 point). We did not conduct a rich content analysis to see if the documents correctly or thoroughly discussed these 15 dimensions of scientific merit. We only looked to see if the concepts were mentioned at all. If two reviewers disagreed about the relevance of a mentioned keyword, this was scored as 0.5.

## RESULTS

The results of our investigation into the guidance materials from 35 U.S.-based research IRBs are presented in Table 1. All of our 15 dimensions of scientific merit were mentioned at least once across this cohort of IRB materials.

We found that most (> 89%) IRB guidance materials included some mention of concepts that we related to a basic understanding what is being tested in a clinical trial, and on whom. Specifically, the keywords for study design (99%), subject recruitment (90%), intervention justification (97%), and intervention risk (97%) were all frequently mentioned. Less frequently did materials contain mention of concepts related to the importance of study location (84%), duration (86%), inclusion of diverse populations (79%), intervention administration (61%).

Less frequently still were the concepts more closely related to the study’s quality, or its capacity to produce an informative, valuable scientific result—not merely its description. Into this category we would group the keywords for comparative treatment (70%), randomization (63%), primary/secondary endpoints (71%), and subject withdrawal (80%).

We found that only about half the time did materials any mention of bias reduction (53%) and outcome measurement tools (57%). Guidance for controlling confounders was by far the least often mentioned (10%) of our dimensions.

## DISCUSSION

Our investigation reveals that IRB guidance materials vary in the comprehensiveness across these 15 dimensions of scientific merit. Elements related to the study population were generally covered, whereas guidance for more technical elements relating to bias, confounders, and outcomes was often lacking. This later point is discouraging, since the more technical dimensions of a trial’s protocol would prima facie be the dimensions that would benefit the most from thorough guidance. But indeed, if IRB applicants and members are not given comprehensive instructions for how to thoroughly assess scientific merit, then it should not be surprising to see that so many scientifically deficient clinical trials receive IRB approval.

We should emphasize as well that we only looked for *mentions* of the keywords—not a thorough content analysis. One the one hand, this can be considered a limitation of our investigation, since guidance may mention “placebo,” for example, without providing any substantive guidance for the IRB as to how a placebo control should be assessed with respect to the protection of human subjects’ interests. On the other hand, the liberality of our extraction and analysis (i.e., giving “credit” to IRB materials for merely including a keyword) makes the absence of any of these dimensions all the more striking—and troubling.

Indeed, the success of evidence-based medicine, and the improvements in healthcare that are expected to flow therefrom, requires the volunteer efforts of patients, the insights of investigators, and the resources of sponsors to conduct clinical trials that produce reliable information. IRB approval is meant to assure the routine ethical alignment of these parties and their interests. The plethora of uninformative clinical trials suggests that IRB review practices are falling short in this essential ethical task. Our results shed further light on why this may be, as well as identifying a concrete opportunity for immediate improvement: Guidance materials for IRB members can be strengthened by including at least some discussion of all of our 15 dimensions.
